# *Nascer no Brasil*: continuity of care during pregnancy and postpartum period for women and newborns

**DOI:** 10.11606/s1518-8787.2020054002021

**Published:** 2020-10-23

**Authors:** Sonia Duarte de Azevedo Bittencourt, Elenice Machado Cunha, Rosa Maria Soares Madeira Domingues, Barbara Almeida Soares Dias, Marcos Augusto Bastos Dias, Jacqueline Alves Torres, Maria do Carmo Leal

**Affiliations:** I Fundação Oswaldo Cruz Escola Nacional de Saúde Pública Sérgio Arouca Departamento de Epidemiologia e Métodos Quantitativos em Saúde Rio de JaneiroRJ Brasil Fundação Oswaldo Cruz . Escola Nacional de Saúde Pública Sérgio Arouca . Departamento de Epidemiologia e Métodos Quantitativos em Saúde . Rio de Janeiro , RJ , Brasil; II Fundação Oswaldo Cruz Escola Politécnica de Saúde Joaquim Venâncio Laboratório de Educação Profissional em Vigilância em Saúde Rio de JaneiroRJ Brasil Fundação Oswaldo Cruz . Escola Politécnica de Saúde Joaquim Venâncio . Laboratório de Educação Profissional em Vigilância em Saúde (LAVSA/EPSJV). Rio de Janeiro , RJ , Brasil; III Fundação Oswaldo Cruz Instituto Nacional de Infectologia Evandro Chagas Laboratório de Pesquisa Clínica em DST/Aids Rio de JaneiroRJ Brasil Fundação Oswaldo Cruz . Instituto Nacional de Infectologia Evandro Chagas . Laboratório de Pesquisa Clínica em DST/Aids . Rio de Janeiro , RJ , Brasil; IV Fundação Oswaldo Cruz Escola Nacional de Saúde Pública Sérgio Arouca Programa de Pós-Graduação em Epidemiologia em Saúde Pública Rio de JaneiroRJ Brasil Fundação Oswaldo Cruz . Escola Nacional de Saúde Pública Sérgio Arouca . Programa de Pós-Graduação em Epidemiologia em Saúde Pública . Rio de Janeiro , RJ , Brasil; V Fundação Oswaldo Cruz Instituto Nacional de Saúde da Mulher, da Criança e do Adolescente Fernandes Figueira Rio de JaneiroRJ Brasil Fundação Oswaldo Cruz . Instituto Nacional de Saúde da Mulher, da Criança e do Adolescente Fernandes Figueira . Rio de Janeiro , RJ , Brasil; VI Agência Nacional de Saúde Suplementar Rio de JaneiroRJ Brasil Agência Nacional de Saúde Suplementar . Rio de Janeiro , RJ , Brasil

**Keywords:** Maternal and Child Health Services, Prenatal care Pregnancy Postpartum period, Brazilian Unified Health System, Epidemiological Surveys

## Abstract

**OBJECTIVE:**

To estimate the adequacy of health care during pregnancy and the postpartum period in puerperal women and newborn users of the Unified Health System and verify the factors associated with greater adequacy.

**METHODS:**

We used data obtained in the hospital interview, the prenatal card and the first telephone interview of 12,646 women participating in the study *Nascer no Brasil* (Birth in Brazil), conducted in 2011 and 2012. In the first stage of the analysis, the sociodemographic and obstetric characteristics of women and the estimation of adequacy of prenatal and postpartum care indicators are described. In the second stage, the cascade of care for actions related to puerperal women and their newborns is presented. Finally, maternal factors associated with the adequacy of the line of care are verified by means of multiple logistic regression.

**RESULTS:**

Only two of the four prenatal indicators were considered satisfactory: initiation up to the 16th week of pregnancy and adequate number of appointments. The guidance on which maternity to go for delivery, as well as the guidance to perform the puerperal appointment and the performance of the heel prick test have reached partial level of adequacy. The puerperal appointment, the first routine appointment of the newborn and the obtaining of the heel prick test results presented unsatisfactory adequacy. In the joint analysis of indicators regarding the effective use of services, only 1.5% of mothers and their babies received all recommended health care. Multiparous women living in the North, Northeast and Midwest, with lower schooling, presented the lowest chances of continuity of care.

**CONCLUSIONS:**

The indicators evaluated indicate that almost all women and their children presented partial and disjointed care, showing that the coordination of care is still a challenge in the health care of women and children in the puerperal pregnancy period.

## INTRODUCTION

The puerperal period, known for its association with vulnerability in women and children, has been contemplated with strategies aimed at expanding access and use of health services for the protection of this population. Among such strategies, we seek the articulation of services, in order to build care networks to guarantee the continuity of care ^1^ .

Continuity of care is a multifaceted concept that in the international literature generally refers to individual care in the field of primary care but is also understood in the sphere of health service management. According to Reid et al. ^2^ and Haggerty et al. ^3^ , this concept presents two essential elements: the existence of a real bond between the patient and the primary care physician or team, and continuity defined as a line of care that requires coordination.

In the postulates of Starfield ^4^ , continuity of care is discussed in the scope of care coordination and operates with the underlying idea of uninterrupted succession of the health care process to a given problem. It refers to the ability of the health system to organize services regarding clinical records and the personnel responsible for care, in addition to the users’ perception of the extent of care.

Regarding service management, the Joint Commission on Accreditation of Healthcare Organizations ^5^ recognizes continuity as one of the measurable dimensions of quality of care and defines it as “the degree to which patient care is coordinated between professionals or providers over time.”

We worked under the premise that continuity of care should be maintained in the puerperal period and, as such, needs to be evaluated. Therefore, the objective of the article is to estimate the adequacy of the line of care during pregnancy and the puerperal period in both mother and child users of the Unified Health System (SUS) as indicative of the continuity of care, verifying the factors associated with the greatest adequacy.

## METHODS

The national hospital-based study *Nascer no Brasil* , conducted between 2011 and 2012, evaluated prenatal, delivery and postpartum care of women who had as pregnancy outcome a newborn alive with any weight or gestational age (GA), or a dead fetus weighing more than 500 grams or GA greater than 22 weeks.

The sample was selected in three stages. In the first, hospitals with more than 500 deliveries per year were stratified according to the five macro-regions of the country, location (capital or countryside) and type of service (public, mixed or private). Then 266 hospitals with probability of selection proportional to the number of deliveries in each of the strata were selected. In the second stage, the number of days needed to interview 90 puerperal women in each hospital was defined using an inverse sampling method. In the third, eligible women were selected on each day of fieldwork. A total of 23,894 women were interviewed.

Data collection included face-to-face interviews conducted during hospitalization; extraction of data from the prenatal card, when available; extraction of data from maternal and newborn medical records after hospital discharge; and two telephone interviews after hospital discharge. More information about the sampling process and design of the study *Nascer no Brasil* can be found at Vasconcellos et al. ^6^ and Leal et al. ^7^

In this analysis, we used data obtained in the hospital interview, in the prenatal card and in the first telephone interview, conducted between 43 and 365 days after delivery (median 81 days, interquartile interval 55–117 days) with 12,646 women (68% in relation to puerperal women interviewed immediately).

We considered eligible for this analysis the puerperal women who had no previous pregnancy, used public services for delivery care and had hospital discharge up to 15 days after delivery, whose outcome was a full-term, living newborn with discharge in less than seven days. Twin pregnancies, premature newborns and newborns who remained hospitalized for more than seven days were excluded due to the need for differentiated care. Puerperal women whose pregnancy outcome was stillbirth or neonatal death were excluded, as they did not use the entire line of care.

The following indicators were considered based on recommendations from the Ministry of Health (MoH):

1. Initiation of prenatal care up to the 16th gestational week.
2. Adequate number of appointments for GA in childbirth.
3. Prenatal guidance on reference service for delivery care.
4. Guidance provided during hospitalization regarding attendance to a health service to perform the postpartum appointment.
5. Conducting postpartum appointment in the first fifteen days after delivery.
6. Application of the BCG vaccine in newborns.
7. Application of the first dose of hepatitis B vaccine on newborns.
8. Performance of the heel prick test in the first seven days of life.
9. Conducting the first routine appointment of the newborn in the first week of life.
10. Results of the heel prick test in the first month of life.

Indicators 1 and 2, related to prenatal care, were obtained primarily from prenatal card data, using maternal information when the card was not available (30% of the cases). All the others were obtained through the maternal report. For indicators 1 and 2, the recommendations of the Brazilian MoH in force at the time of the study were used ^8^ . Indicators 3 and 4 correspond to integration activities between different levels of the system, with indicator 3 referring to the connection of pregnant women to the reference maternity, regulated by law since 2007 ^9^ . The other indicators refer to health actions performed after delivery.

Data analysis was performed in three stages. The first consisted of a descriptive analysis of the sociodemographic and obstetric characteristics of the women included in this analysis and the estimation of adequacy of prenatal and postpartum care indicators with their respective 95% confidence intervals (95%CI). The adequacy calculation considered the proportion of women who answered “yes” in relation to the total number of eligible women. As assessment criteria, the range of values contained in the 95%CI between 75 and 100% was considering “satisfactory,” between 50 and 74% as “partial” and below 50% as “unsatisfactory.” In the second stage, the cascades of care for actions related to the care of mothers and newborns were made. In this type of analysis, the adequacy of each item is calculated as a proportion of those who answered “yes” in relation to the total who received the previous stage of care. For the indicators 5 to 9 of the newborn care cascade, 9 (i.e., the first routine appointment of the newborn in the first week of life) was considered the last item, since indicators 6, 7 and 8 could be performed both during hospitalization and after discharge.

Finally, maternal factors associated with the adequacy of the line of care are verified by means of multiple logistic regression. As an outcome variable, a minimum indicator of the use of prenatal and postpartum care services was used. Factors considered as minimum indicators were: if the pregnant woman started prenatal care up to the 16th gestational week and received the appropriate number of appointments for gestational age at delivery; if the puerperal mother had received a postpartum appointment up to 15 days after delivery; if the child received the BCG vaccine and hepatitis B vaccine, had performed the heel prick test, attended the first routine childcare appointment in the first week of life and received the result of the heel prick test in the first month of life. Indicators 1 and 2 were related to maternity-issued guidance regarding the delivery and the puerperal appointment, and thus were not included in this minimum indicator as they did not reflect the use of these services (considering that the lack of guidance would not be an obstacle in seeking delivery care and puerperal appointment).

Explanatory variables used were: region of residence (North, Northeast, Southeast, South or Midwest), maternal age (< 20, 20 to 34 or ≥ 35 years), self-reported skin color (white, brown or black), maternal education (some elementary school, elementary school, high school or college), marital status (live or not with a partner), employment (yes or no), parity (first child or not), satisfaction with current pregnancy (satisfied, partially satisfied, or dissatisfied) and diagnosis of previous chronic disease (women with reports of chronic hypertension, non-gestational diabetes mellitus, heart disease, severe anemia/hemoglobinopathy, asthma, lupus, hyperthyroidism, chronic kidney disease, seizures, stroke, or liver disease). Women with self-reported yellow and indigenous skin color were excluded from this analysis due to its reduced quantity (1.5% of the total).

The univariate analysis estimated the crude odds ratios and 95%CI. All variables with significance level < 0.20 were included in the multiple model, remaining in the final model those with p < 0.05 values. The results of the final model were expressed as adjusted odds ratios with their corresponding 95%CI.

All statistical analyses were performed through the statistical program SPSS version 17.0 by weighting and calibrating the data and incorporating the design effect, considering the complex sampling process. The weighting aimed to deal with losses in the telephone interview, which were higher than 30%. The justification for applying weights regarding the lack of response is the assumption that non-respondents would have provided similar answers, on average, to those of the interviewees for each stratum and adjustment category. Further details on the weighting and calibration procedure used can be obtained from Vasconcellos et al. ^6^

The study *Nascer no Brasil* was approved by the Research Ethics Committee of the Sergio Arouca National School of Public Health, Oswaldo Cruz Foundation (ENSP/Fiocruz), opinion no. 92/2010. All measures were carried out in order to ensure the secrecy and confidentiality of the information. Before each interview, consent was obtained after reading the free and informed consent form.

## RESULTS

A total of 16,220 women were part of this analysis. The Southeast (41.7%) and Northeast (29.4%) regions concentrated most births. Most women (70.7%) were between 20 and 34 years old and 21.4% were adolescents. The skin color declared by most was brown (60.1%), followed by white (29.1%) and black (9.4%). Approximately one quarter of the women reported having only incomplete elementary school and only 3.2% reported higher education. Most women lived with their partner (79.8%) and 34.8% were employed. Almost half (46%) were primiparous and, among puerperal women, 66.7% reported having been satisfied with pregnancy, while one third declared some degree of dissatisfaction. The proportion of women who had some chronic disease before pregnancy was 7.8% ( Table 1 ).

**Table 1:** Distribution of sociodemographic, obstetric and current pregnancy characteristics. Brazil, 2011–2012.

Sociodemographic characteristics	n	%
Region		
Southeast	6,757	41.7
South	1,903	11.7
Midwest	1,059	6.5
Northeast	4,769	29.4
North	1,732	10.7
Age		
35 years or older	1,294	8.0
20 to 34 years	11,463	70.7
12 to 19 years	3,464	21.4
Ethnicity		
White	4,713	29.1
Brown	9,752	60.1
Black	1,518	9.4
Yellow	173	1.1
Indigenous	63	0.4
Schooling level		
College or graduate degree	518	3.2
High school	6,289	38.9
Middle school	4,895	30.3
Some middle school	4,463	27.6
Marital status		
With a partner	12,925	79.8
Without partner	3,268	20.2
Employment situation		
Yes	5,646	34.8
No	10,572	65.2
Characteristics of current pregnancy		
First child		
Yes	7,457	46.0
No	8,763	54.0
Vaccination during pregnancy		
Satisfied	10,765	66.7
Moderately satisfied	3,761	23.3
Unsatisfied	1,616	10.0
History of chronic disease		
No	14,955	92.2
Yes	1,265	7.8
Total	16,220	100.0

The analysis of the indicators ( Table 2 ) revealed that 74.8% of women started prenatal care in up to 16 weeks of pregnancy, and a similar proportion (75.2%) was able to carry out the appropriate number of appointments for GA, with both recommendations reaching the satisfactory level of adequacy. Guidance on which maternity to seek for the delivery (57%) and guidance to perform the puerperal appointment (66.5%) have reached partial level of adequacy. The postpartum appointment was performed by 32.2% of the women, with unsatisfactory adequacy.

**Table 2:** Description of indicators related to continuity of care in women who were users of the Unified Health System. Brazil, 2011–2012.

Indicators of continuity of care	n	%	95%CI
Initiation of prenatal care up to the 16th gestational week.	11,987	74.8	73.3–76.3
Adequate number of appointments for GA	11,790	75.2	73.5–76.7
Guidance on which hospital, maternity or birthing center to look for	9,231	57.0	54.3–59.5
Guidance to attend the health service to carry out the childbirth revision appointment	10,732	66.5	64.0–68.8
Postpartum appointment in the first 15 days after delivery	10,827	32.2	30.1–34.3
Newborn vaccinated with BCG	16,023	99.1	98.9–99.3
Heel prick test in the first week of life	9,761	60.2	57.4–62.9
Newborn vaccinated against hepatitis B	15,541	96.1	94.9–97.0
First routine appointment of the newborn in the first week of life.	2,953	18.2	16.7–19.9
Obtaining of the heel prick test results in the first month of life.	3,789	23.4	21.1–25.8
Total	16,220	100.0	100.0–100.0

GA: gestational age; NB: newborn; 95%CI: 95% confidence interval.

Application of the BCG vaccine was the indicator with the highest proportion of adequacy (99.1%), followed by vaccination with the first dose against hepatitis B (96.1%), both achieving satisfactory adequacy. The performance of the heel prick test was observed in 60.2% of newborns, reaching a partial level of adequacy. The performance of the first routine appointment of the newborn and the results of the heel prick test reached the lowest proportions, respectively 18.2% and 23.4%, both indicators with unsatisfactory adequacy.

**Figure 1:**
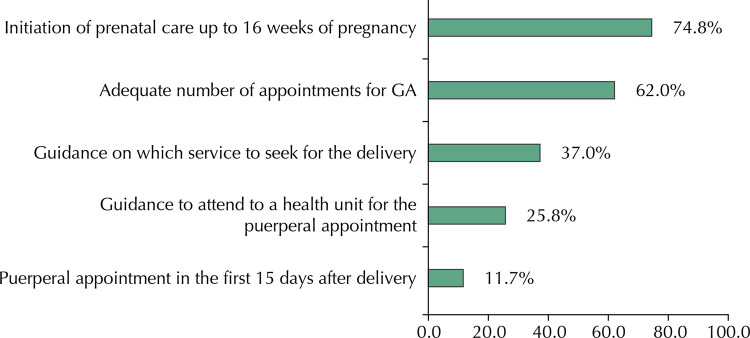
Cascade of adequacy for continuity of care in women. Brazil, 2011–2012.

The cascade of care in women in pregnancy and puerperium ( Figure 1 ) showed that, of the total number of women who started prenatal care in up to 16 weeks, 62% had an adequate number of appointments according to GA, and in this group 37% were instructed on which service to seek for delivery care. Among them, 25.8% were instructed to attend to a health service to perform the postpartum appointment; of these, only 11.7% were able to perform it.

GA: Gestational age

**Figure 2:**
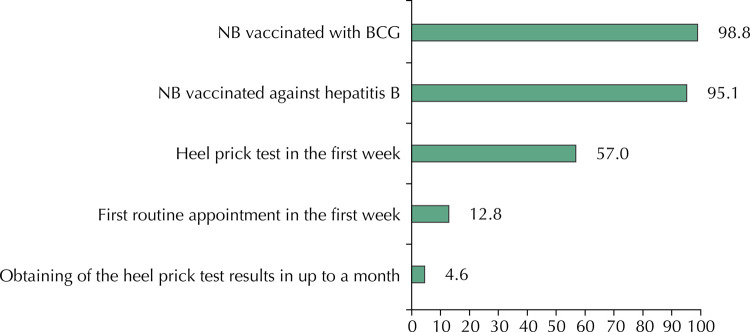
Cascade of adequacy for continuity of care in newborns. Brazil, 2011–2012.

The cascade of care of newborns ( Figure 2 ) showed that almost all of those who received BCG were also vaccinated against hepatitis B. Of those who received both vaccines, only 57% underwent the heel prick test; of these, 12.8% had their first routine appointment. In this group, only 4.6% obtained the heel prick test result up to the first month of life, managing to perform all the recommended actions. In the joint analysis of indicators regarding the effective use of services, only 1.5% of mothers and their babies received all recommended health care.

NB: newborn

Table 3 shows the result of logistic regression of sociodemographic, obstetric and current pregnancy characteristics associated with continuity of care. The univariate analysis showed a lower chance of continuity of care in women living in the Northeast (odds ratio [OR] = 0.10; 95%CI 0.05–0.22), North (OR = 0.13; 95%CI 0.03–0.55) and Midwest regions (OR = 0.50; 95%CI 0.26–0.94); who declared themselves brown-skinned (OR = 0.52; 95%CI 0.33-0.83); with less schooling, prevailing those with complete elementary school (OR = 0.39; 95%CI 0.20–0.77) and incomplete elementary school (OR = 0.22; 95%CI 0.11–0.45); who were unemployed (OR = 0.54; 95%CI 0.33–0.90); had previous births (OR = 0.64; 95%CI 0.47–0.86); and were dissatisfied with pregnancy (OR = 0.51; 95%CI 0.27–0.97). In the multiple analysis, schooling, parity and region of residence maintained a significant association with the adequacy of continuity of care. Women living in the North, Northeast and Midwest regions had the lowest chances of adequacy, with the North and Northeast regions presenting seven to ten times greater chance of inadequate care than the South region. There is also a gradient of adequacy according to schooling, with a directly proportional decrease, and the chance of inadequacy is almost four times higher in women with incomplete elementary school compared with those with higher education.

**Table 3:** Distribution of sociodemographic, obstetric and current pregnancy characteristics. Brazil, 2011–2012.

		Composite indicator ^a^					
	% (95%CI)	Crude OR	95%CI	p	Adjusted OR	95%CI	p
Sociodemographic characteristics							
Region							
Southeast	2.1 (1.4–3.1)	0.59	0.34–1.05	< 0.001	0.58	0.32–1.05	< 0.001
Midwest	1.7 (1.0–2.9)	0.50	0.26–0.94		0.46	0.24–0.89	
Northeast	0.4 (0.2–0.7)	0.10	0.05–0.22		0.10	0.05–0.22	
North	0.5 (0.1–1.8)	0.13	0.03–0.55		0.13	0.03–0.54	
South	3.4 (2.4–4.9)	1.00			1.00		
Age							
35 years or older	1.2 (0.6–2.4)	0.71	0.34–1.48	0.460	-	-	-
12 to 19 years	1.3 (0.7–2.4)	0.79	0.47–1.35		-	-	-
20 to 34 years	1.7 (1.3–2.1)	1.00			-	-	-
Ethnicity							
Brown	1.2 (0.9–1.7)	0.52	0.33–0.83	0.013	-	-	-
Black	1.3 (0.7–2.6)	0.57	0.29–1.10		-	-	-
White	2.3 (1.6–3.3)	1.00			-	-	-
Schooling level							
High school	2.0 (1.5–2.7)	0.56	0.31–1.01	< 0.001	0.57	0.31–1.06	0.002
Middle school	1.4 (0.8–2.4)	0.39	0.20–0.77		0.41	0.21–0.82	
Some middle school	0.8 (0.5–1.2)	0.22	0.11–0.45		0.27	0.13–0.55	
College or graduate degree	3.5 (2.0–6.1)	1.00			1.00		
Marital status							
Without partner	1.7 (0.9–2.9)	1.10	0.61–1.99	0.741	-	-	-
With a partner	1.5 (1.2–2.0)	1.00			-	-	-
Employment situation							
No	1.2 (0.8–1.7)	0.54	0.33–0.90	0.017	-	-	-
Yes	2.2 (1.5–3.1)	1.00			-	-	-
Characteristics of current pregnancy							
First child							
No	1.2 (0.9–1.6)	0.64	0.47–0.86	0.004	0.67	0.49–0.92	0.014
Yes	1.9 (1.4–2.6)	1.00			1.00		
Vaccination during pregnancy							
Unsatisfied	0.9 (0.5–1.7)	0.51	0.27–0.97	0.034	-	-	-
Moderately satisfied	1.2 (0.8–2.0)	0.70	0.45–1.10		-	-	-
Satisfied	1.8 (1.3–2.3)	1.00			-	-	-
History of chronic disease ^b^							
No	1.5 (1.2–2.0)	1.05	0.61–1.82	0.852	-	-	-
Yes	1.5 (0.9–2.5)	1.00			-	-	-
Total	1.5 (1.2–2.0)						

95%CI: 95% confidence interval; OR: odds ratio

^a^ Initiation of prenatal care until the 16th gestational week, adequate number of appointments for GA, postpartum appointment in the first 15 days postpartum, newborn vaccinated with BCG, newborn vaccinated against hepatitis B, foot test in the first week of life, routine appointment of the NB in the first week of life and result of the foot test until the first month of life.

^b^ Postpartum women with the following conditions: hypertension, diabetes mellitus, heart disease, severe anemia or hemoglobinopathy, asthma, lupus, hyperthyroidism, chronic kidney disease, seizures, stroke or liver disease.

## DISCUSSION

Out of the ten evaluated indicators for use of health services, four presented satisfactory adequacy, three presented partial adequacy and three presented unsatisfactory adequacy. However, when evaluated jointly for both mother and baby, only 1.5% of the puerperal women and their newborns followed the minimal recommended actions for continuity of care during pregnancy and postpartum.

The beginning of the prenatal care was evaluated as adequate, considering the 16-week parameter, recommended at the time of the study ^8^ . However, the current recommendation of *Rede Cegonha* ^1^ (RC) is the initiation of prenatal care up to the 12th gestational week. By this parameter, the adequacy would be partial. Similarly, the minimum number of six appointments, also recommended at the time of the study, was expanded in more recent protocols, and more than seven appointments are currently recommended for a common pregnancy ^1 , 10^ . Both late initiation of prenatal care and insufficient amount of contact with the health service favored the fragmentation of care and the incompleteness of the recommended procedures, affecting the effectiveness of prenatal care in the prevention of negative outcomes ^11^ .

Although the connection of pregnant women to the reference maternity for childbirth care has been regulated in the country since 2007 ^9^ , 43% of women were not connected to the service, being subject to the availability of hospital beds. This situation may be related to the low articulation between prenatal, childbirth and postpartum care services, and probably contributed to the expansion of inequities in access to services, besides compromising the effectiveness of health care for this particular group.

The percentage of puerperal appointment was low, around 30%. This was the worst-performing indicator of maternal care, thus compromising the integrality of care. Among other objectives, the puerperal appointment aims to evaluate the occurrence of complications after delivery, support breastfeeding and advise on contraception and baby care, besides detecting important changes ^12^ , such as postpartum depression ^13 , 14^ , estimated at 26.3% among the puerperal women interviewed in the study *Nascer no Brasil* ^15^ .

Regarding the indicators for continuity of care in newborns, those related to immunization presented the best percentages of adequacy. The rate of BCG vaccination reported was close to that observed in a household survey conducted in Brazilian capitals in 2006 ^16^ . The hepatitis B vaccine also presented satisfactory coverage for the dose given at birth. These results reflect the recognition of the importance of immunization, resulting both from the time of implementation and consolidation of the National Immunization Program ^17^ and, more recently, from the Bolsa Família program, since vaccination is one of the conditions for the family’s permanence in the program ^18^ .

The performance of the heel prick test in the first week of life presented partial adequacy, with a percentage similar to that observed in the database of the National Newborn Screening Program ^19^ . Factors such as partial adequacy in data collection at the recommended time and the unsatisfactory adequacy of the obtaining of results until the first month of life were observed in less than a quarter of newborns. This reflects delays in the different phases of the screening process, which may negate the benefits of early detection ^20^ .

Finally, the low rate of attendance for the routine appointment in the first week of life, reported by only 12.8% of women, may be associated with the maternal perception that it would only be necessary in the presence of a disease and not for regular monitoring of child development ^17^ . Geographical, financial and organizational barriers ^21^ , as well as the lack of a social support network, can also contribute to the non-implementation of the appointment.

The lower percentages of adequacy of continuity of care observed among women living in the North and Northeast, regions of the country with worse economic and social development, reflect the lower availability of physical resources and use of health services ^22 , 23^ , despite investments and improvement of some health indicators in recent years. Regarding socioeconomic characteristics, women with lower schooling level had a lower percentage of adequacy, evidencing social inequality. As for the worst result of adequacy among multiparous women, it is likely related to the difficulty in attending the service due to lack of social support in the care of other children or to the feeling of having sufficient knowledge and experience, thus attributing less importance to care in this period ^24^ .

The results of this study refer to women hospitalized for deliveries in hospitals with more than 500 deliveries per year. Although these hospitalizations correspond to 80% of the pregnant women in the country, it is likely that those not included in the analyses, such as those who gave birth at home or in hospitals with lower volume of delivery, as well as twin newborns, those who evolved to neonatal death or those at high risk who remained hospitalized for more than seven days, present a different profile regarding use of health services in the study period. Regarding the reliability of the information reported by the women, it is important to highlight the possibility of memory bias, especially because of the long timespan between the interview conducted in the hospital and telephone contact.

This study, by analyzing data from the line of care of the puerperal pregnancy period, gave visibility to the existing weaknesses in the continuity of care of pregnant women, puerperal women and newborn users of SUS. The indicators evaluated show that almost all women and their children experienced partial and disjointed care, indicating that the coordination of care is still a challenge in the health care of women and children in the pregnancy and puerperal period. These findings may partially explain the persistence of still unfavorable perinatal outcomes.

The determinants of the use of health services include both the adequate provision of these services and the perception of the need for care ^25^ . The findings of this study, in isolation, do not allow us to state where such gap is; whether in the supply, coordination and articulation between the services, or perception of the need on the part of women. However, studies on continuity of care ^3^ allow us to conclude that the therapeutic bond with professionals in primary care, who in turn perform the function of coordination of care, together with the existence of health services articulated in care networks, would be predominant in ensuring the adequacy of care.

Finally, we should highlight recent national initiatives to improve care for the group in question, especially *Rede Cegonha* ^1^ . Such projects foster the implementation of a new health care model, in which women’s health needs become the basis for the development of the line of care, in order to structure the articulation of the responsibilities and functions of each health service involved in prenatal, childbirth and puerperal care of women and newborns, thus ensuring comprehensive, timely and resolutive care. Further studies are needed to evaluate the effects of these actions on indicators of use and continuity of care.
