# Supplementary material for: Nascer no Brasil: continuity of care during pregnancy and postpartum period for women and newborns
Source: Rev Saude Publica. 2020 Oct 23;54:100. doi: 10.11606/s1518-8787.2020054002021 (PMC7584409; doi:10.11606/s1518-8787.2020054002021)
Supplement: Translation

# Translation: Nascer no Brasil: continuidade do cuidado na gestação e pós-parto à mulher e ao recém-nato

**Authors:** Sonia Duarte de Azevedo Bittencourt, Elenice Machado Cunha, Rosa Maria Soares Madeira Domingues, Barbara Almeida Soares Dias, Marcos Augusto Bastos Dias, Jacqueline Alves Torres, Maria do Carmo Leal

**Affiliations:** I Brasil Fundação Oswaldo Cruz. Escola Nacional de Saúde Pública Sérgio Arouca. Departamento de Epidemiologia e Métodos Quantitativos em Saúde. Rio de Janeiro, RJ, Brasil; II Brasil Fundação Oswaldo Cruz. Escola Politécnica de Saúde Joaquim Venâncio. Laboratório de Educação Profissional em Vigilância em Saúde (LAVSA/EPSJV). Rio de Janeiro, RJ, Brasil; III Brasil Fundação Oswaldo Cruz. Instituto Nacional de Infectologia Evandro Chagas. Laboratório de Pesquisa Clínica em DST/Aids. Rio de Janeiro, RJ, Brasil; IV Brasil Fundação Oswaldo Cruz. Escola Nacional de Saúde Pública Sérgio Arouca. Programa de Pós-Graduação em Epidemiologia em Saúde Pública. Rio de Janeiro, RJ, Brasil; V Brasil Fundação Oswaldo Cruz. Instituto Nacional de Saúde da Mulher, da Criança e do Adolescente Fernandes Figueira. Rio de Janeiro, RJ, Brasil; VI Brasil Agência Nacional de Saúde Suplementar. Rio de Janeiro, RJ, Brasil

**Keywords:** Serviços de Saúde Materno-Infantil, Cuidado Pré-Natal, Gravidez, Período Pós-Parto, Sistema Único de Saúde, Inquéritos Epidemiológicos

## Abstract

**OBJETIVO:**

Estimar a adequação da linha de cuidado da atenção à saúde durante a gestação e o pós-parto em puérperas e recém-natos usuários do Sistema Único de Saúde e verificar os fatores associados à maior adequação.

**MÉTODOS:**

Foram utilizados os dados obtidos na entrevista hospitalar, no cartão de pré-natal e na primeira entrevista telefônica de 12.646 mulheres participantes do estudo Nascer no Brasil, realizado em 2011 e 2012. Na primeira etapa da análise, descrevem-se as características sociodemográficas e obstétricas das mulheres e a estimativa de adequação de indicadores de cuidado pré-natal e pós-parto. Na segunda etapa, apresenta-se a cascata de cuidados das ações relativas ao cuidado da mulher e do recém-nascido. Por último, verificam-se os fatores maternos associados à adequação da linha de cuidado, por meio de regressão logística múltipla.

**RESULTADOS:**

Apenas dois dos quatro indicadores do pré-natal foram considerados satisfatórios: início até a 16ª semana de gestação e número adequado de consultas. Atingiram patamar parcial de adequação a orientação sobre qual maternidade procurar para ter o parto, a orientação para comparecer ao serviço de saúde para realizar a consulta de puerpério e a realização do teste do pezinho. A consulta de puerpério, a primeira consulta de rotina do recém-nascido e o recebimento do teste do pezinho apresentaram adequação insatisfatória. Na análise conjunta dos indicadores que dizem respeito à efetiva utilização dos serviços, apenas 1,5% das mães e seus bebês receberam todos os cuidados em saúde recomendados. Mulheres residentes no Norte, Nordeste e Centro-Oeste, com menor escolaridade e multíparas apresentaram as menores chances de continuidade do cuidado.

**CONCLUSÕES:**

Os indicadores avaliados sinalizam que quase a totalidade das mulheres e seus filhos apresentaram uma assistência parcial e desarticulada, indicando que a coordenação do cuidado ainda é um desafio na atenção à saúde de mulheres e crianças no período gravídico puerperal.

## INTRODUÇÃO

O período gravídico puerperal, de reconhecida vulnerabilidade para mulher e criança, vem sendo contemplado com estratégias que visam a ampliar acesso e utilização de serviços de saúde para a proteção dessa população. Dentre as estratégias, busca-se a articulação de serviços, no intuito de formar redes de atenção com vias à garantia da continuidade do cuidado ^1^ .

A continuidade do cuidado é um conceito multifacetado que na literatura internacional geralmente refere-se ao cuidado individual no domínio da atenção primária, mas que também é compreendido na esfera do gerenciamento dos serviços de saúde. Para Reid et al. ^2^ e Haggerty et al. ^3^ , esse conceito apresenta dois elementos essenciais: a existência de um vínculo real entre o paciente e o médico ou equipe da atenção primária e a continuidade definida como uma linha de cuidados que requer coordenação.

Nos postulados de Starfield ^4^ , a continuidade do cuidado é discutida no escopo da coordenação do cuidado, e tem subjacente a ideia da sucessão ininterrupta do processo da atenção à saúde para determinado problema de saúde. Remete à capacidade do sistema de saúde de organizar os serviços no que concerne aos registros clínicos e ao pessoal responsável pelos atendimentos, além da percepção dos usuários sobre a extensão da atenção.

No que alude à esfera do gerenciamento dos serviços, a *Joint Commission on Accreditation of Healthcare Organizations* ^5^ reconhece a continuidade como uma das dimensões mensuráveis da qualidade do cuidado e a define como “o grau em que o cuidado ao paciente é coordenado entre os profissionais ou entre os prestadores ao longo do tempo”.

Partindo da premissa de que a continuidade do cuidado, em sua interface com o conceito de utilização dos serviços de saúde, deve ser almejada no período gravídico puerperal e, como tal, precisa ser avaliada, o objetivo do artigo é estimar a adequação da linha de cuidado da atenção à saúde durante a gestação e o pós-parto em puérperas e recém-natos usuários do Sistema Único de Saúde (SUS) como indicativo da continuidade do cuidado e verificar os fatores associados à maior adequação.

## MÉTODOS

O estudo nacional de base hospitalar Nascer no Brasil, realizado entre 2011 e 2012, avaliou a assistência pré-natal, ao parto e ao pós-parto de mulheres que tiveram como desfecho da gestação um recém-nato vivo com qualquer peso ou idade gestacional (IG), ou um feto morto com peso maior que 500 gramas ou IG maior que 22 semanas.

A amostra foi selecionada em três etapas. Na primeira, os hospitais com mais de 500 partos por ano foram estratificados de acordo com as cinco macrorregiões do país, localização (capital ou interior) e tipo de serviço (público, misto ou privado), sendo selecionados 266 hospitais com probabilidade de seleção proporcional ao número de partos em cada um dos estratos. Na segunda etapa, o número de dias necessários para entrevistar 90 puérperas em cada hospital foi definido utilizando um método de amostragem inversa. Na terceira, foram selecionadas as mulheres elegíveis em cada dia do trabalho de campo. Foram entrevistadas 23.894 mulheres.

A coleta de dados incluiu entrevista face a face realizada durante a internação hospitalar; extração de dados do cartão de pré-natal, quando disponível; extração de dados dos prontuários maternos e do recém-nato (RN) após a alta hospitalar; e duas entrevistas telefônicas após a alta hospitalar. Mais informações sobre o processo amostral e desenho do estudo Nascer no Brasil podem ser obtidas em Vasconcellos et al. ^6^ e Leal et al. ^7^

Na presente análise foram utilizados os dados obtidos na entrevista hospitalar, no cartão de pré-natal e na primeira entrevista telefônica, realizada entre 43 e 365 dias após o parto (mediana 81 dias, intervalo interquartil 55–117 dias) com 12.646 mulheres (68% em relação às puérperas entrevistadas no pós-parto imediato).

Foram consideradas elegíveis para esta análise as puérperas com gestação única, financiamento público da atenção ao parto e alta hospitalar até 15 dias após o parto, cujo desfecho foi um RN a termo, vivo e com alta hospitalar antes de sete dias de vida. Gestações gemelares, RN prematuros e RN que permaneceram internados por mais de sete dias foram excluídos devido à necessidade de cuidados diferenciados. Puérperas cujo desfecho da gestação tenha sido um natimorto ou neomorto foram excluídas pela não utilização de toda a linha de cuidado de atenção.

Foram considerados os seguintes indicadores baseados em recomendações do Ministério da Saúde (MS):

1. Início da assistência pré-natal (PN) até a 16ª semana gestacional.
2. Número adequado de consultas para a IG no parto.
3. Orientação no pré-natal sobre serviço de referência para atenção ao parto.
4. Orientação fornecida durante a internação para o parto sobre comparecimento a um serviço de saúde para realizar a consulta de puerpério.
5. Realização de consulta de puerpério nos primeiros quinze dias pós-parto.
6. Vacinação do RN com BCG.
7. Vacinação do RN com a primeira dose da vacina contra hepatite B.
8. Realização do teste do pezinho nos primeiros sete dias de vida.
9. Realização da primeira consulta de rotina do RN na primeira semana de vida.
10. Recebimento do resultado do teste do pezinho no primeiro mês de vida.

Os indicadores 1 e 2, relativos ao cuidado pré-natal, foram obtidos prioritariamente a partir de dados de cartão de pré-natal, sendo utilizada a informação materna quando o cartão não estava disponível (30% dos casos). Todos os demais foram obtidos por meio do relato materno. Para os indicadores 1 e 2, foram utilizadas as recomendações do MS brasileiro vigentes na época do estudo ^8^ . Os indicadores 3 e 4 correspondem às atividades de integração entre diversos níveis do sistema, sendo o indicador 3, referente à vinculação da gestante à maternidade de referência, regulamentado por lei desde 2007 ^9^ . Os demais indicadores referem-se às ações de saúde realizadas após o parto.

A análise dos dados foi feita em três etapas. A primeira consistiu em análise descritiva das características sociodemográficas e obstétricas das mulheres incluídas nessa análise e a estimativa de adequação dos indicadores de cuidado pré-natal e pós-parto com seus respectivos intervalos de confiança de 95% (IC95%). O cálculo da adequação foi realizado considerando-se a proporção de mulheres que respondeu “sim” em relação ao total de elegíveis e, como critério de julgamento, a faixa de valores contidos nos IC95% entre 75 e 100% como “satisfatório”, entre 50 e 74% como “parcial” e abaixo de 50% como “insatisfatório”.

Na segunda etapa foram efetuadas as cascatas de cuidados das ações relativas ao cuidado da mulher e do RN. Nesse tipo de análise, a adequação de cada item é calculada como proporção das que responderam “sim” em relação ao total da que receberam a etapa anterior do cuidado. Para os indicadores de 5 a 9 da cascata de cuidados do RN, o 9 (realização da primeira consulta de rotina do RN na primeira semana de vida) foi considerado o último item, posto que os indicadores 6, 7 e 8 poderiam ser realizados tanto durante a internação hospitalar como após a alta.

Por último, verificaram-se os fatores maternos associados à adequação da linha de cuidado por meio de regressão logística múltipla. Como variável desfecho, empregou-se um indicador mínimo de utilização de serviços de atenção ao pré-natal e ao pós-parto. Foi considerado como indicador mínimo: a gestante ter iniciado a assistência pré-natal até a 16 ^a^ semana gestacional e ter recebido o número adequado de consultas para a idade gestacional no parto; a puérpera ter recebido consulta de puerpério até 15 dias após o parto; o RN ter recebido a vacina BCG e a vacina contra a hepatite B, ter realizado o teste do pezinho, ter recebido a primeira consulta de rotina da puericultura na primeira semana de vida e ter recebido o resultado do teste do pezinho no primeiro mês de vida. Os indicadores 1 e 2, relacionados às orientações recebidas sobre a maternidade de referência para o parto e sobre a consulta de puerpério, não foram incluídos nesse indicador mínimo por não refletirem a utilização dos serviços, pois o não recebimento dessas orientações não seria impeditivo para o acesso à assistência ao parto e à consulta de puerpério.

Como variáveis explicativas foram incluídas a região de residência da mulher (Norte, Nordeste, Sudeste, Sul ou Centro-Oeste), idade materna (< 20, 20 a 34 ou ≥ 35 anos), cor da pele autorreferida (branca, parda ou preta), escolaridade materna (fundamental incompleto, fundamental completo, médio, ou superior ou maior), situação conjugal (vive ou não com o companheiro), trabalho remunerado (sim ou não), paridade (primípara ou não), satisfação com a gestação atual (satisfeita, mais ou menos satisfeita, ou insatisfeita) e diagnóstico de doença crônica anterior (mulheres com relato de hipertensão arterial crônica, diabetes mellitus não gestacional, doença cardíaca, anemia grave/hemoglobinopatia, asma, lúpus, hipertireoidismo, doença renal crônica, convulsões, acidente vascular cerebral ou doença hepática). Mulheres com cor da pele autorreferida amarela e indígenas foram excluídas desta análise por seu reduzido quantitativo (1,5% do total).

A análise univariada estimou as razões de chance não ajustadas e os IC95%. Todas as variáveis com nível de significância < 0,20 foram incluídas no modelo múltiplo, permanecendo no modelo final aquelas com valor de p < 0,05. Os resultados do modelo final foram expressos como razões de chance ajustadas com seus correspondentes IC95%.

Toda a análise estatística foi realizada por meio do programa estatístico SPSS versão 17.0, com utilização de ponderação e calibração dos dados e incorporação do efeito de desenho, considerando o processo complexo da amostragem. A ponderação visou lidar com as perdas na entrevista telefônica, que foram superiores a 30%. A justificativa para aplicação de pesos de não resposta é a suposição de que os não respondentes teriam fornecido respostas semelhantes, em média, às dos entrevistados para cada estrato e categoria de ajuste. Maiores detalhes sobre o procedimento de ponderação e calibração utilizados podem ser obtidos em Vasconcellos et al. ^6^

O estudo Nascer no Brasil foi aprovado pelo Comitê de Ética em Pesquisa da Escola Nacional de Saúde Pública Sergio Arouca, Fundação Oswaldo Cruz (ENSP/Fiocruz), parecer nº 92/2010. Todos os cuidados foram adotados visando garantir o sigilo e confidencialidade das informações. Antes da realização de cada entrevista, foi obtido consentimento após leitura do termo de consentimento livre e esclarecido.

## RESULTADOS

Um total de 16.220 mulheres fizeram parte desta análise. As regiões Sudeste (41,7%) e Nordeste (29,4%) concentraram a maior parte dos nascimentos. A maioria das mulheres (70,7%) tinha idade entre 20 e 34 anos e 21,4% eram adolescentes. A cor da pele declarada pela maioria foi parda (60,1%), seguida pelas brancas (29,1%) e pretas (9,4%). Aproximadamente um quarto das mulheres referia ter apenas o ensino fundamental incompleto e apenas 3,2% informou nível superior de escolaridade. A grande maioria das mulheres vivia com o companheiro (79,8%) e 34,8% tinham trabalho remunerado. Quase metade (46%) eram primíparas e, entre o total das puérperas, 66,7% declararam terem ficado satisfeitas com a gestação, enquanto um terço declarou algum grau de insatisfação. A proporção de mulheres que apresentava alguma doença crônica anterior à gestação foi de 7,8% ( Tabela 1 ).

**Tabela 1:** Distribuição das características sociodemográficas, obstétricas e da gestação atual. Brasil, 2011–2012.

Características sociodemográficas	n	%
Região		
Sudeste	6.757	41,7
Sul	1.903	11,7
Centro-Oeste	1.059	6,5
Nordeste	4.769	29,4
Norte	1.732	10,7
Idade		
35 anos ou mais	1.294	8,0
20 a 34 anos	11.463	70,7
12 a 19 anos	3.464	21,4
Raça/cor		
Branca	4.713	29,1
Parda	9.752	60,1
Preta	1.518	9,4
Amarela	173	1,1
Indígena	63	0,4
Escolaridade		
ES completo ou maior	518	3,2
EM completo	6.289	38,9
EF completo	4.895	30,3
EF incompleto	4.463	27,6
Situação conjugal		
Com companheiro	12.925	79,8
Sem companheiro	3.268	20,2
Trabalho remunerado		
Sim	5.646	34,8
Não	10.572	65,2
Características da gestação atual		
Primípara		
Sim	7.457	46,0
Não	8.763	54,0
Satisfação na gestação		
Satisfeita	10.765	66,7
Mais ou menos satisfeita	3.761	23,3
Insatisfeita	1.616	10,0
Doença crônica anterior		
Não	14.955	92,2
Sim	1.265	7,8
Total	16.220	100,0

ES: ensino superior; EM: ensino médio: EF: ensino fundamental.

A análise dos indicadores ( Tabela 2 ) revelou que 74,8% das mulheres iniciaram o pré-natal até a 16 ^a^ semana de gestação, e proporção semelhante (75,2%) conseguiu realizar o número adequado de consultas para a IG, com ambas recomendações atingindo o nível satisfatório de adequação. Atingiram patamar parcial de adequação a orientação sobre qual maternidade procurar para ter o parto (57%) e a orientação para comparecer ao serviço de saúde para realizar a consulta de puerpério (66,5%). A consulta de puerpério foi realizada por 32,2% das mulheres, tendo adequação insatisfatória.

**Tabela 2:** Descrição dos indicadores relacionados à continuidade do cuidado em mulheres usuárias do Sistema Único de Saúde. Brasil, 2011–2012.

Indicadores da continuidade do cuidado	n	%	IC95%
Início da assistência pré-natal até a 16ª semana gestacional	11.987	74,8	73,3–76,3
Número adequado de consultas para a IG	11.790	75,2	73,5–76,7
Orientada sobre qual hospital, maternidade ou casa de parto procurar para ter o parto	9.231	57,0	54,3–59,5
Orientada a comparecer ao serviço de saúde para realizar a consulta de revisão do parto	10.732	66,5	64,0–68,8
Consulta de puerpério nos primeiros 15 dias pós-parto	10.827	32,2	30,1–34,3
RN vacinado com a BCG	16.023	99,1	98,9–99,3
Teste do pezinho na primeira semana de vida	9.761	60,2	57,4–62,9
RN vacinado contra hepatite B	15.541	96,1	94,9–97,0
Primeira consulta de rotina do RN na primeira semana de vida	2.953	18,2	16,7–19,9
Resultado do teste do pezinho até o primeiro mês de vida	3.789	23,4	21,1–25,8
Total	16.220	100,0	100,0–100,0

IG: idade gestacional; RN: recém-nascido; IC95%: intervalo de confiança de 95%.

Vacinar o bebê com a BCG foi o indicador com a maior proporção de realização (99,1%), seguido pela vacinação com a primeira dose contra hepatite B (96,1%), ambos alcançando adequação satisfatória. A realização do teste do pezinho foi observada em 60,2% dos recém-nascidos, atingindo nível parcial de adequação. A realização da primeira consulta de rotina do RN e o recebimento do resultado do teste do pezinho atingiram as menores proporções, respectivamente 18,2% e 23,4%, ambos indicadores com adequação insatisfatória.

A cascata das ações recomendadas visando à continuidade do cuidado em mulheres na gestação e puerpério ( Figura 1 ) mostrou que, do total de mulheres que iniciaram o pré-natal até a 16 ^a^ semana, 62% tiveram um número adequado de consultas de acordo com a IG, e nesse grupo 37% foram orientadas sobre qual serviço procurar para assistência ao parto. Dentre elas, 25,8% foram orientadas a comparecer a um serviço de saúde para realizar a consulta de puerpério; dessas, apenas 11,7% conseguiram realizá-la.

**Figura 1:** Cascata da adequação da continuidade do cuidado em mulheres. Brasil, 2011–2012.

IG: idade gestacional

A cascata de cuidados dos recém-natos ( Figura 2 ) mostrou que quase a totalidade daqueles que receberam BCG também foram vacinados contra hepatite B. Dos que receberam as duas vacinas, apenas 57% realizaram o teste do pezinho; desses, 12,8% tiveram a primeira consulta de rotina. Nesse grupo, apenas 4,6% obtiveram o resultado do teste do pezinho até o primeiro mês de vida, conseguindo realizar todas as ações recomendadas. Na análise conjunta dos indicadores que dizem respeito à efetiva utilização dos serviços, apenas 1,5% das mães e seus bebês receberam todos os cuidados em saúde recomendados.

**Figura 2:** Cascata da adequabilidade da continuidade do cuidado em recém-nascidos. Brasil, 2011–2012.

RN: recém-nascido

A Tabela 3 apresenta o resultado da regressão logística das características sociodemográficas, obstétricas e da gestação atual associadas à continuidade do cuidado. A análise univariada mostrou menor chance de continuidade do cuidado nas mulheres que moram nas regiões Nordeste ( *odds ratio* [OR] = 0,10; IC95% 0,05–0,22), Norte (OR = 0,13; IC95% 0,03–0,55) e Centro-Oeste (OR = 0,50; IC95% 0,26–0,94); que se declararam de cor da pele parda (OR = 0,52; IC95% 0,33–0,83); com menor escolaridade, prevalecendo o ensino fundamental completo (OR = 0,39; IC95% 0,20–0,77) e o ensino fundamental incompleto (OR = 0,22; IC95% 0,11–0,45); sem trabalho remunerado (OR = 0,54; IC95% 0,33–0,90); multíparas (OR = 0,64; IC95% 0,47–0,86); e insatisfeitas com a gestação (OR = 0,51; IC95% 0,27–0,97). Na análise múltipla, escolaridade, paridade e região de residência mantiveram associação significativa com a adequação da continuidade do cuidado. Mulheres residentes nas regiões Norte, Nordeste e Centro-Oeste apresentaram as menores chances de adequação, com as regiões Norte e Nordeste apresentando chance sete a dez vezes maior de cuidado inadequado do que a região Sul. Observa-se também um gradiente de adequação segundo a escolaridade, com diminuição diretamente proporcional, sendo a chance de inadequação quase quatro vezes maior em mulheres com ensino fundamental incompleto em relação àquelas com nível superior.

**Tabela 3:** Características sociodemográficas, obstétricas e da gestação atual associadas a continuidade do cuidado. Brasil, 2011–2012.

		Indicador composto ^a^					
	% (IC95%)	OR bruta	IC95%	p	OR ajustada	IC95%	p
Características sociodemográficas							
Região							
Sudeste	2,1 (1,4–3,1)	0,59	0,34–1,05	< 0,001	0,58	0,32–1,05	< 0,001
Centro–Oeste	1,7 (1,0–2,9)	0,50	0,26–0,94		0,46	0,24–0,89	
Nordeste	0,4 (0,2–0,7)	0,10	0,05–0,22		0,10	0,05–0,22	
Norte	0,5 (0,1–1,8)	0,13	0,03–0,55		0,13	0,03–0,54	
Sul	3,4 (2,4–4,9)	1,00			1,00		
Idade							
35 anos ou mais	1,2 (0,6–2,4)	0,71	0,34–1,48	0,460	-	-	-
12 a 19 anos	1,3 (0,7–2,4)	0,79	0,47–1,35		-	-	-
20 a 34 anos	1,7 (1,3–2,1)	1,00			-	-	-
Raça/cor							
Parda	1,2 (0,9–1,7)	0,52	0,33–0,83	0,013	-	-	-
Preta	1,3 (0,7–2,6)	0,57	0,29–1,10		-	-	-
Branca	2,3 (1,6–3,3)	1,00			-	-	-
Escolaridade							
EM completo	2,0 (1,5–2,7)	0,56	0,31–1,01	< 0,001	0,57	0,31–1,06	0,002
EF completo	1,4 (0,8–2,4)	0,39	0,20–0,77		0,41	0,21–0,82	
EF incompleto	0,8 (0,5–1,2)	0,22	0,11–0,45		0,27	0,13–0,55	
ES completo ou maior	3,5 (2,0–6,1)	1,00			1,00		
Situação conjugal							
Sem companheiro	1,7 (0,9–2,9)	1,10	0,61–1,99	0,741	-	-	-
Com companheiro	1,5 (1,2–2,0)	1,00			-	-	-
Trabalho remunerado							
Não	1,2 (0,8–1,7)	0,54	0,33–0,90	0,017	-	-	-
Sim	2,2 (1,5–3,1)	1,00			-	-	-
Características da gestação atual							
Primípara							
Não	1,2 (0,9–1,6)	0,64	0,47–0,86	0,004	0,67	0,49–0,92	0,014
Sim	1,9 (1,4–2,6)	1,00			1,00		
Satisfação na gestação							
Insatisfeita	0,9 (0,5–1,7)	0,51	0,27–0,97	0,034	-	-	-
Mais ou menos satisfeita	1,2 (0,8–2,0)	0,70	0,45–1,10		-	-	-
Satisfeita	1,8 (1,3–2,3)	1,00			-	-	-
Doença crônica anterior ^b^							
Não	1,5 (1,2–2,0)	1,05	0,61–1,82	0,852	-	-	-
Sim	1,5 (0,9–2,5)	1,00			-	-	-
Total	1,5 (1,2–2,0)						

ES: ensino superior; EM: ensino médio: EF: ensino fundamental; IC95%: intervalo de confiança de 95%; OR: *odds ratio*

^a^ Início da assistência pré-natal até a 12ª semana gestacional, número adequado de consultas para a IG, consulta de puerpério nos primeiros 15 dias pós-parto, RN vacinado com a BCG, RN vacinado contra hepatite B, teste do pezinho na primeira semana de vida, consulta de rotina do RN na primeira semana de vida e resultado do teste do pezinho até o primeiro mês de vida.

^b^ Puérperas com as seguintes condições: hipertensão, diabetes mellitus, doença cardíaca, anemia grave ou hemoglobinopatia, asma, lúpus, hipertireoidismo, doença renal crônica, convulsões, acidente vascular cerebral ou doença hepática.

## DISCUSSÃO

Dos dez indicadores de utilização de serviços de saúde avaliados, quatro apresentaram adequação satisfatória, três adequação parcial e três adequação insatisfatória. Entretanto, quando avaliados conjuntamente para o binômio mãe-bebê, apenas 1,5% das puérperas e RN tiveram a utilização mínima das ações recomendadas para a continuidade do cuidado durante a gestação e o pós-parto.

O início do acompanhamento PN foi avaliado como adequado, considerando-se o parâmetro de 16 semanas, recomendado à época do estudo ^8^ . Entretanto, a recomendação atual da Rede Cegonha ^1^ (RC) é o início do acompanhamento PN até a 12ª semana gestacional. Adotando esse parâmetro, a adequação seria parcial. Da mesma forma, o número mínimo de seis consultas, também recomendado à época do estudo, foi ampliado em protocolos mais recentes, sendo atualmente recomendadas mais de sete consultas para uma gestação de risco habitual ^1 , 10^ . O início tardio do acompanhamento PN e um número insuficiente de contatos com o serviço de saúde favorecem a fragmentação do cuidado e a incompletude dos procedimentos preconizados, afetando a efetividade da assistência pré-natal na prevenção de desfechos negativos ^11^ .

Embora a vinculação das gestantes à maternidade de referência para a atenção ao parto esteja regulamentada no país desde 2007 ^9^ , 43% das mulheres não foram vinculadas, ficando sujeitas à busca de disponibilidade de leito hospitalar para a realização do parto. Essa situação pode estar relacionada à baixa articulação entre os serviços de atenção ao pré-natal, parto e puerpério, e provavelmente contribuiu para ampliar as iniquidades no acesso, além de comprometer a efetividade da assistência à saúde do grupo em questão.

O percentual de realização da consulta puerperal foi baixo, em torno de 30%. Esse foi o indicador de pior desempenho do cuidado materno, comprometendo a integralidade da assistência. Dentre outros objetivos, a consulta de puerpério visa avaliar a ocorrência de complicações após o parto, apoiar o aleitamento materno e orientar sobre contracepção e cuidados com o bebê, além de detectar importantes alterações ^12^ , como a depressão pós-parto ^13 , 14^ , estimada em 26,3% entre as puérperas entrevistadas no estudo Nascer no Brasil ^15^ .

Quanto aos indicadores da continuidade do cuidado dos recém-nascidos, os relativos à imunização obtiveram os melhores percentuais de adequação. A vacinação BCG informada foi próxima à observada em inquérito domiciliar realizado nas capitais brasileiras em 2006 ^16^ . A vacina contra hepatite B apresentou também cobertura satisfatória para a dose ministrada ao nascer. Esses resultados refletem o reconhecimento da importância da imunização, decorrente tanto do tempo de implantação e consolidação do Programa Nacional de Imunização ^17^ e, mais recentemente, do programa Bolsa Família, uma vez que a vacinação é uma das condicionalidades para permanência da família no programa ^18^ .

A realização do teste do pezinho na primeira semana de vida apresentou adequação parcial, com percentual similar ao observado na base de dados do Programa de Triagem Neonatal ^19^ . A adequação parcial da coleta na época recomendada e a adequação insatisfatória do recebimento do resultado até o primeiro mês de vida, observadas em menos de um quarto dos RN, refletem atrasos nas diferentes fases do processo de triagem, situação que pode anular os benefícios da detecção precoce ^20^ .

Por fim, o baixo comparecimento para a consulta de rotina na primeira semana de vida, relatado por apenas 12,8% das mulheres, pode estar associado à percepção materna de que só seria necessário na presença de doença e não para acompanhamento do desenvolvimento infantil ^17^ . Também podem contribuir para a não concretização da consulta as barreiras geográficas, financeiras e organizacionais ^21^ , bem como a falta de uma rede de apoio social.

Os menores percentuais de adequação da continuidade do cuidado observados entre as mulheres residentes no Norte e Nordeste, regiões do país com menor desenvolvimento econômico e social, refletem a menor disponibilidade de recursos físicos e utilização de serviços de saúde ^22 , 23^ , apesar dos investimentos e melhoria de alguns indicadores de saúde nos últimos anos. No que tange às características socioeconômicas, as mulheres de menor escolaridade apresentaram menor percentual de adequação, evidenciando a desigualdade social. Quanto ao pior resultado de adequação entre as multíparas, é provável que esteja relacionado à dificuldade no comparecimento ao serviço por falta de suporte social no cuidado dos demais filhos ou ao sentimento de ter conhecimento e experiência suficientes, atribuindo menor importância ao cuidado nesse período ^24^ .

Os resultados deste estudo referem-se às mulheres internadas para realização de partos em hospitais com mais de 500 partos por ano. Embora essas internações correspondam a 80% das gestantes do país, é provável que as não incluídas nas análises apresentadas, como as que pariram no domicílio ou em hospitais com menor volume de parto, assim como os RN gemelares, os que evoluíram para o óbito neonatal ou os de alto risco que permaneceram internados por mais de sete dias, apresentem perfil diferenciado de utilização de serviços de saúde no período estudado. Quanto à confiabilidade das informações referidas pelas mulheres, é importante destacar a possibilidade de viés de memória, sobretudo por conta do amplo intervalo entre a entrevista realizada no hospital e o contato telefônico.

Este estudo, ao analisar dados da linha de cuidado do período gravídico puerperal, deu visibilidade às fragilidades existentes na continuidade do cuidado de gestantes, puérperas e recém-natos usuários do SUS. Os indicadores avaliados sinalizam que a quase totalidade das mulheres e seus filhos apresentaram uma assistência parcial e desarticulada, indicando que a coordenação do cuidado ainda é um desafio na atenção à saúde de mulheres e crianças no período gravídico puerperal. Esses achados podem explicar parcialmente a persistência de resultados perinatais ainda desfavoráveis.

Os determinantes da utilização de serviços de saúde incluem tanto a oferta adequada desses serviços quanto a percepção da necessidade de cuidado ^25^ . Os achados deste estudo, isoladamente, não permitem afirmar onde está a lacuna, se na oferta, coordenação e articulação entre os serviços que devem compor a linha de cuidado, ou se na percepção da necessidade por parte das mulheres. Contudo, estudos sobre a continuidade do cuidado ^3^ permitem concluir que o vínculo terapêutico com profissionais na atenção primária, que por sua vez exercem a função de coordenação do cuidado, juntamente com a existência de serviços de saúde articulados em redes de atenção, seriam preponderantes na garantia da adequabilidade do cuidado.

Devem-se ressaltar, por fim, as iniciativas nacionais recentes adotadas para melhorar o atendimento ao grupo em questão, em especial a RC ^1^ , para fomentar a implementação de um novo modelo de atenção à saúde, cuja classificação das necessidades de saúde da mulher passa ser a base para o desenvolvimento da linha de cuidado, de modo a estruturar a articulação das responsabilidades e funções de cada serviço de saúde envolvido na assistência ao pré-natal, parto e pós-parto de mulheres e recém-nascidos, e assim garantir o cuidado integral, oportuno e resolutivo. Novos estudos são necessários para avaliar os efeitos dessas inciativas nos indicadores de utilização e de continuidade do cuidado.
